# Addressing the pitfalls when designing intervention studies to discover and validate biomarkers of habitual dietary intake

**DOI:** 10.1007/s11306-019-1532-3

**Published:** 2019-05-02

**Authors:** A. J. Lloyd, N. D. Willis, T. Wilson, H. Zubair, E. Chambers, I. Garcia-Perez, L. Xie, K. Tailliart, M. Beckmann, J. C. Mathers, J. Draper

**Affiliations:** 1 Institute of Biological, Environmental and Rural Sciences, Aberystwyth University, Aberystwyth, SY23 3DA UK; 2 Human Nutrition Research Centre, Institute of Cellular Medicine, Newcastle University, Newcastle-upon-Tyne, NE2 4HH UK; 3 Nutrition and Dietetic Research Group, Division of Diabetes, Endocrinology and Metabolism, Department of Medicine, Hammersmith Hospital Campus, Imperial College London, London, W12 0NN UK

**Keywords:** Dietary biomarkers, Free-living population, Healthy eating policies, High resolution metabolomics

## Abstract

**Introduction:**

Dietary exposure monitoring within populations is reliant on self-reported measures such as Food Frequency Questionnaires and diet diaries. These methods often contain inaccurate information due to participant misreporting, non-compliance and bias. Urinary metabolites derived from individual foods could provide additional objective indicators of dietary exposure. For biomarker approaches to have utility it is essential that they cover a wide-range of commonly consumed foods and the methodology works in a real-world environment.

**Objectives:**

To test that the methodology works in a real-world environment and to consider the impact of the major sources of likely variance; particularly complex meals, different food formulations, processing and cooking methods, as well as the dynamics of biomarker duration in the body.

**Methods:**

We designed and tested a dietary exposure biomarker discovery and validation strategy based on a food intervention study involving free-living individuals preparing meals and collecting urine samples at home. Two experimental periods were built around three consecutive day menu plans where all foods and drinks were provided (n = 15 and n = 36).

**Results:**

The experimental design was validated by confirming known consumption biomarkers in urinary samples after the first menu plan. We tested biomarker performance with different food formulations and processing methods involving meat, wholegrain, fruits and vegetables.

**Conclusion:**

It was demonstrated that spot urine samples, together with robust dietary biomarkers, despite major sources of variance, could be used successfully for dietary exposure monitoring in large epidemiological studies.

**Electronic supplementary material:**

The online version of this article (10.1007/s11306-019-1532-3) contains supplementary material, which is available to authorized users.

## Introduction

A key factor in effective implementation of public health strategies is the need for validated population-level dietary exposure screening methods with which to determine the effectiveness of ‘healthy eating’ interventions to change eating habits. To date, assessment of dietary exposure has relied on self-reported measures of intake derived using tools such as Food Frequency Questionnaires, dietary recall and diet diaries (Penn et al. 2010). However these methods have well understood limitations as a result of misreporting and bias, and depend upon food composition tables for estimation of intakes of energy, nutrients and other food constituents (Bingham et al. 1994). These limitations may be overcome, at least in part, by the demonstration that metabolites derived from specific foods or food groups present in urine samples provide biomarkers of dietary exposure (Lee et al. 2006; Lovegrove et al. 2004; Marklund et al. 2010). Urinary dietary exposure biomarkers are based on the concept that urinary excretion of specific metabolite(s) reflects, quantitatively, intake of a corresponding food or nutrient over a fixed period of time. In some cases, multi-metabolite biomarker panels may provide more reliable estimation of dietary exposure than a single-biomarker approach (reviewed by (Garcia-Aloy et al. 2017)). To add value to the assessment of habitual dietary intake, a dietary biomarker panel should contain markers for a wide range of commonly consumed foods and these markers should be specific and sensitive. In addition, given the genotype-dependent inter-individual differences in metabolism of some food-derived components (van Duynhoven et al. 2011), biomarkers panels should be based on metabolites that are universally applicable, where possible. The ideal biomarker is highly specific for one food item or food group, is not detected in the biological sample of interest when the specific food item is not ingested, and shows a distinct dose- and time- dependent response following consumption (Kristensen et al. 2012). At present, biomarker panels are not sufficiently comprehensive or well-validated to replace traditional dietary assessment methods and so, in practice, they should be used in combination with self-report methods to improve the accuracy of dietary intake measurement (Potischman and Freudenheim 2003).

Several approaches have been used for the discovery of dietary biomarkers in urine. For example, in well-controlled dietary intervention studies, participants consume a single test food in isolation in a single meal or repeated meals and baseline and post prandial spot urine samples are collected over a few hours (Edmands et al. 2011; Lloyd et al. 2011b). Following this approach, we used non-targeted metabolomics in combination with rapid metabolite fingerprinting methods to identify a range of potential biomarker candidates for widely consumed foods that are relatively abundant and easily detected in urine (Fave et al. 2011; Lloyd et al. 2011a; Lloyd et al. b; Primrose et al. 2011). In addition, using self-reported dietary data, participants in cohort studies can be classified into consumers and non/low consumers of a particular food/food group and metabolite profiles/fingerprints of representative bio-banked urine samples used to identify distinguishing metabolites which are assumed to be characteristic of the particular food/food group (Garcia-Aloy et al. 2015; Gibbons et al. 2015). Lastly, attempts have been made to derive metabolic profiles/fingerprints that reflect dietary patterns rather than single foods/food groups (Garcia-Perez et al. 2017). With further development and validation, the latter approach could have wide utility but it’s associated with greater conceptual and practical challenges. These approaches are reviewed by (Gibbons and Brennan 2017). Whilst these metabolomic-based approaches have led to the discovery of dietary biomarkers for several foods, current approaches and study designs have multiple limitations.

For dietary biomarkers to have significant utility in the implementation of future public health policies, their performance in real-world environments needs to be demonstrated. In real-life situations, foods are not usually consumed in isolation but rather as part of potentially complex meals in which they are co-consumed with other foods which may attenuate the ability to identify and to validate potential biomarkers of dietary exposure. In addition, different food preparation, processing and cooking methods may affect the stability, availability and biotransformation fate and, therefore concentration in urine, of metabolites that are potential food intake biomarkers. Further, the future utility of any biomarker panel will be enhanced greatly if its coverage of commonly-consumed foods is comprehensive. Currently, chemical biomarkers are available for only a relatively small number of specific foods and food components and most are of uncertain validity (Lee et al. 2006; Lovegrove et al. 2004; Marklund et al. 2010). There are many foods and food groups of high public health relevance for which dietary exposure biomarkers are not yet available. Thus there is a need to populate dietary biomarker panels with new biomarkers for a wider range of food/food groups, to enable them to provide independent, objective, and accurate estimates of dietary exposure.

The Metabolomics at Aberystwyth, Imperial and Newcastle (MAIN) Study was designed to address some of these challenges. Here we describe the design and testing of a dietary exposure biomarker discovery strategy based on a comprehensive food intervention study that mimicked an annual eating pattern using commonly consumed foods as recorded by the National Diet and Nutrition Survey (NDNS) (Department of Health 2012). Free-living individuals prepared and consumed the test foods and collected urine samples at home. We aimed to test the utility of this approach for efficient characterization of recent dietary exposure in free-living individuals sampled with minimal intrusion on normal daily activities.

## Experimental methods

### Ethical approval

A favorable ethical opinion was obtained following Proportionate Review by the East Midlands—Nottingham 1 National Research Ethics Committee (14/EM/0040). The trial was adopted into the UK Clinical Research Network (CRN) Portfolio (16037) and is registered with International Standard Randomized Controlled Trials Number (ISRCTN), 88921234. All participants gave written informed consent, and the study was carried out in accordance with the Declaration of Helsinki.

### Study design and urine sampling

The MAIN Study at Newcastle comprises two interrelated food interventions (Willis et al. Submitted), the first of which (Lloyd et al. Submitted) validated a food intervention strategy designed to mimic an annual eating pattern in a home setting over a period of a few weeks and additionally developed methods for collecting spot urine samples suitable for deployment of biomarker measurement technology in free-living populations. The present manuscript focuses largely on the second intervention which explores the impact of portion size and food formulation or cooking method on biomarker performance. In brief, the project included two controlled food intervention studies in free-living populations who consumed the test foods within two three-day menu plans, equating to six different menus. We selected foods/food groups for menus using information on the top 2–3 most highly consumed foods within food groups and used the most common preparation methods for these foods, as recorded by the NDNS years 1–3 (Department of Health 2012), together with Public Health England policy advice from The Eatwell Plate (Food Standards Agency 2009) which has now been revised to The Eatwell Guide (Public Health England 2016). We used standard portion sizes based on the UK Food Standards Agency (Food Standards Agency 1994) or manufacturers’ suggestions.

In the first study we employed a cross-over design in which, on the day before the study (Pre-day), participants were randomized to either a standardized evening meal (Supplemental data 1a) or a low polyphenol evening meal of their choosing (avoiding the foods listed in Supplemental data 1b). In the following week, participants were crossed-over to the alternative pre-evening meal. The standardized evening meal was designed to minimize urine metabolome variability as the meal contained only food secondary metabolites likely to be digested and excreted without modification or excreted as well-described bio transformed metabolites, which were unaffected by the individuals’ gut microflora or general genetic disposition. Menu plans were followed on experimental days 1, 2 and 3 during which all foods and drinks were provided for participants in the portion sizes appropriate for UK conventional meals (breakfast, lunch, afternoon snack and dinner). These foods/drinks were prepared, cooked and consumed by participants at home. The Post-day was the day following completion of the 3-day menu plan, when the last biological samples were collected. The pre-determined times for urine collection are described in (Lloyd et al. Submitted), but included post dinner (bed-time), first morning void (FMV), fasting and post breakfast and post lunch urine samples on each experimental day including the morning of the post-day. Participants collected urine samples in a plastic jug, recorded the volume together with the date, time and type of sample and transferred an aliquot to a labelled sterile 25 mL Universal tube. This sample was placed in an opaque cool bag, in the fridge at 4 °C. When transferred to the laboratory by the participant, further aliquots (2 mL) of all urine samples were made and were stored (Eppendorf tubes) at − 80 °C.

### Urine sample preparation, extraction and adjustment

To take into account relative differences in fluid intake between participants all urine samples were normalized by refractive index prior to analysis to ensure all MS measurements were made within a similar dynamic range. Refractive index normalization takes the total sample composition into account when normalising and not just the concentration of a single analyte. Refractive index normalization was selected over volume and creatinine normalization, as urine volumes are not always recorded successfully with spot samples and additionally it has been shown that there may be a gender bias present in urine creatinine concentrations (Ulaszewska et al. 2019). Samples were defrosted overnight in a 4 °C fridge. Once defrosted, samples were centrifuged (600×*g* for 5 min at 4 °C), placed on ice and aliquots of thawed urine (1000 µL) was transferred into labelled 2 mL Eppendorf tubes. The remaining samples were returned to a − 20 °C freezer. An OPTI Hand Held Refractometer (Bellingham Stanley™ Brix 54 Model) was calibrated with de-ionised water (dH_2_O) and dried with tissue according to the manufacturer’s instruction. Following this 220 µL of sample was transferred onto the refractometer dish, the specific gravity (SG) value was recorded in triplicate and temperature was noted. The refractometer was rinsed with dH_2_O between samples and dried with tissue. Average SG values were calculated. Based on these figures, aliquots of the required amounts of urine from centrifuged 2 mL Eppendorf tubes and dH2O were transferred into new 2 mL Eppendorf tubes on ice to make up 500 µL for extraction. Pre-chilled (− 20 °C) H_2_O: MeOH (3:7) was added to each adjusted sample and vortexed before being refrigerated at 4 °C overnight, ready for analysis.

### Flow infusion-high resolution fingerprinting (FIE-HRMS), multivariate modelling, classification and feature selection

We used a Thermo Exactive (Orbitrap) Mass Spectrometer (MS), equipped with an electrospray ionization source (ESI) and coupled to a Thermo Accela ultra-performance liquid chromatograph. Extracted urine samples were delivered to the electrospray source via a flow solvent (mobile phase) of pre-mixed HPLC grade MeOH (Fisher Scientific) and ultra-pure H_2_O (18.2 Ω) at a ratio of 7:3. The flow rate was 200 μL/min for the first 1.5 min, and 600 μL/min for the remainder of the run. MS signals were collected from 55.000 to 1000.000 *m/z* and from 63.000 to 1000.00 *m/z* for positive and negative mode respectively. RAW files were converted to mzML open file format and centroided (Martens et al. 2011). Dimensionality reduction of the acquired mass spectra was performed by taking each *m/z* value from scans about the apex of the infusion profile and binning the *m/z* and intensity values at 0.01 amu intervals, allowing direct comparison of urine fingerprints, prior to signal annotation. This method has been described in detail elsewhere (Lloyd et al. Submitted; Wilson et al. Submitted)).

Supervised Random Forest (RF) classification was implemented using the randomForest package in R (R Core Team 2013). For all Random Forest models, the number of trees (*ntree)* used was 1000 and the number of variables considered at each internal node (mtry) was the square root of the total number of variables. RF margins of classification and area under the receiver operator characteristic (ROC) curve (AUC) were used to evaluate the performance of classification models, as described previously (Enot et al. 2008). Models were deemed adequate if RF margins were > 0.2 and AUC values were > 0.8, thresholds that we have implemented in previous publications (Enot et al. 2008; Lloyd et al. 2016).

Signals discriminating between sample classes were selected using the following procedure. The fasting and the FMV sample of each experimental day was used as the baseline comparison with the subsequent post-prandial urine samples (post-breakfast, post-lunch and post-dinner) of that day to detect metabolite signals associated with exposure to food items consumed at particular times that day. In addition, for the post-dinner sample the subsequent fasting and the FMV (collected on the following day) was used as an additional comparison to allow detection of longer-duration markers, such as those derived from metabolism of the colonic microbiota and metabolites with longer half-lives e.g. because of temporary sequestration in tissues before excretion via the kidney. To reveal potential explanatory signals responsible for discriminating between baseline and post-prandial urine samples, a combination of RF, AUC and Student’s *t* test, (Enot et al. 2008) was employed. RF feature selection was performed by calculating Importance Scores, calculated as the mean decrease in accuracy over all classes when a feature was omitted from the data. AUC used the area under curve of the sensitivity (true-positive rate) against the specificity (false-positive rate) and Student’s *t* test ranked the features by the absolute value of the relevant P-values. The following thresholds were implemented to confidently identify discriminatory signals and metabolites: RF Importance scores > 0.002, P-values < 0.05, AUC 0.9. Lower thresholds indicated putative discriminatory biomarkers (RF Importance scores > 0.001 < 0.002, AUC > 0.8 < 0.9) and deemed worthy of investigating further. Randomized re-sampling strategies using bootstrapping were applied in the process of feature selection to counteract the effect of any unknown, structured variance in the data. We used 100 bootstraps in pair-wise comparisons for each of the applied statistical operations with 2/3 of data as a training set and the remaining1/3 as the test set. RF was set to ntree = 1000 for each bootstrap which is adequate considering the dimensionality of data.

For metabolite signal annotation, accurate *m/z* values were extracted from the un-binned matrix to enable direct identification of metabolites at 1–5 ppm in the first pass profile. These were queried using MZedDB, an interactive accurate mass annotation tool used to annotate signals by means of neutral loss and/or adduct formation rules (Draper et al. 2009). Ultra High Performance Liquid Chromatography-High Resolution MS (UHPLC-HRMS) and Tandem mass spectrometry (MS^n^) were used for further structural identification of putative biomarkers as described elsewhere (Lloyd et al. 2017). Samples were analyzed on an Exactive Orbitrap (Thermo Scientific) mass spectrometer, coupled to an Accela Ultra UHPLC system (Thermo Scientific). Metabolites were annotated to Metabolomics Standards Initiative (MSI) level 1 (Sumner et al. 2007) if they have matching masses, MS^n^ and retention times with authentic standards or the respective aglycone (if the biotransformation product was unavailable) run under the same conditions. MSI level 2 was used for putative matching compounds without standards (based upon physicochemical properties, retention times and spectral similarity) reported in public/commercial spectral libraries (Lipid Maps, HMDB, Metlin and Massbank (Horai et al. 2010; Sana et al. 2008; Sud et al. 2007; Wishart et al. 2009)). MSI level 3 indicated a putatively characterized compound class (e.g. based upon characteristic physicochemical properties of a chemical class of compounds, or by spectral similarity to known compounds of a chemical class).

### Quantification using TSQ quantum ultra triple quadrupole (QQQ) technology

Quantitative analyses were performed on a TSQ Quantum Ultra triple quadrupole (QQQ) mass spectrometer (Thermo Scientific), equipped with an electro-spray ionization (ESI) source and coupled to an Accela UHPLC system (Thermo Scientific) following a protocol from (Wei et al. 2010). Targeted metabolites together with column HILIC and RP-C18 column chemistries, limit-of-detection (LOD) and limit-of-quantification (LOQ) values are shown in Supplemental data 2. Mass spectra were acquired in multiple reaction monitoring (MRM) mode, in positive and negative ionization mode simultaneously, using optimized values of shimmer offset, collision energy, and tube lens for each MRM transition (see Supplemental data 2 for transitions).

We calculated the mean concentrations of biomarkers in FMV urines the day after consumption and we defined whether the selected food/food group was consumed in a small, medium or large portion by looking at the FSA ‘Food portion sizes’ guide. We denoted a ‘large’ portion as > 1.5 X a suggested FSA medium portion size and a ‘small’ portion size as < 0.5 X a suggested FSA medium portion size (Food Standards Agency 1994).

Mean concentrations of putative biomarkers in FMV urines after consumption of the highest and lowest portion sizes of selected dietary components were compared using the *t* test and correlations between portion size and biomarker concentration were performed using Spearman’s rank correlation.

## Results and discussion

### Utility of published biomarkers to detect dietary exposure in free-living individuals

We first tested whether our flow-infusion high resolution MS fingerprinting biomarker discovery strategy was sufficiently sensitive to detect “known” biomarkers of specific foods when consumed by free-living individuals as components of conventional UK meals prepared and eaten at home. This was achieved by determining whether previously reported urinary biomarkers were highly ranked (i.e. RF Importance scores > 0.002, P-values < 0.05, AUC 0.9) in comparisons between fasting and post-prandial urine samples following acute exposure to specific foods (Table 1 and further signals in Supplemental data 3). Targeted foods included coffee, wholegrain, high-sugar foods, oily fish, cruciferous vegetables, grapes/wine and nuts (described in detail in (Lloyd et al. Submitted)). The majority of these markers appeared discriminatory i.e. detected the presence of the test food. In addition, in most cases, the nature of the meal consumed on the ‘Pre-day’ i.e. either a standardized evening meal (Supplemental data 1a) or a non-standardized low polyphenol meal of the participant’s choice (Supplemental data 1b) did not affect the outcome. Based on RF IS and AUC values (Supplemental data 3), only three putative biomarkers i.e. 5-(3′,4′,5′-trihydroxyphenyl)-γ-valerolactone sulfate, Ferulic acid 4-o-glucuronide and dihydroferulic acid-4-*o*-sulfate were not found to be discriminatory after the non-standardized meal when compared with the standardized meal. However, all three compounds are biotransformation products of other known biomarkers which functioned well and, therefore, these three biomarkers are effectively redundant.

**Table 1:** Validation in a free-living population of previously identified food intake biomarkers

Food or beverage consumed	Biomarker	Ionization products	MSI level and reference
Rye bread	2-Hydroxy-N-(2-hydroxyphenyl)acetamide (HHPAA) glucuronide	[M−H]^1−^	2 (Beckmann et al. 2013; Zhu et al. 2016)
Rye bread	Isopropyl 2-hydroxyphenylcarbamate (benzoxazinoid metabolite)	[M−H]^1−^	2 (Zhu et al. 2016)
Wholegrain/rye bread and coffee	Ferulic acid 4-*o*-sulfate	[M−H]^1−^, [M−SULP−H]^1−^	1 (Bondia-Pons et al. 2013; Edmands et al. 2011)
Wholegrain/rye bread and coffee	Ferulic acid 4-*o*-glucuronide	[M−H]^1−^	1 (Edmands et al. 2011)
Coffee	Caffeine	[M+H]^1+^	1 (Rothwell et al. 2014)
Coffee	Trigonelline (N-methyl nicotinate)	[M+Na]^1+^ and [M+Na]^1+^ ^13^C	1 (Rothwell et al. 2014)
Sweetened-breakfast cereal	Sucrose	[M−H]^1−^, [M+Na]^1+^, [M+K]^1+^	1 (Beckmann et al. 2016)
Salmon	Anserine	[M−H]^1−^, [M+H]^1+^, [M+H]^1+^ ^13^C, [M+Na]^1+^ [M+K]^1+^	1 (Lloyd et al. 2011b)
Salmon	Trimethylamine N-oxide	[2M+H]^1+^	1 (Lloyd et al. 2011b)
Broccoli	S-Methyl-l-cysteine sulfoxide	[M+Na]^1+^	1 (Edmands et al. 2011)
Wine/grapes	Tartaric acid	[M−H]^1−^, [M−H]^1−^ ^13^C, [M+Na−2H]^1−^	1 (Garcia-Perez et al. 2016)
Almonds	4-Hydroxy-5-(3,4-dihydroxyphenyl)-valeric acid	[M−H]^1−^, [M+Na]^1+^	2 (Edmands et al. 2011; Sánchez-Patán et al. 2012)

All markers appeared discriminatory in post-prandial urine collections with the following implemented thresholds: RF Importance scores > 0.001, P-values < 0.05, AUC > 0.8 (see Supplemental data 3 for further details)

*MSI* metabolomics standards initiative

We have reported the use of non-targeted Flow Infusion nominal mass LC–MS fingerprinting of urine samples to identify potential dietary exposure biomarker signals whose structure was confirmed later using targeted high resolution MS/MS fragmentation (Lloyd et al. 2011b). In the current work, for the first time, we report the use of Flow Infusion-High Resolution Fingerprinting (FIE-HRMS) combined with Random Forest feature selection for direct discovery and identification structurally, at high mass accuracy (1–5 ppm), of dietary exposure biomarkers in urine samples. Despite not being specifically targeted for measurement in the present study, these previously published biomarkers proved to discriminate between fasting and post-prandial urine samples from a free-living population, and so help validate our intervention study design for dietary exposure biomarker technology development in a ‘real world’ situation. The considerable inter- and intra-individual variability in human metabolite profiles in urine poses considerable challenges for data normalization in metabolomics studies seeking information on dietary exposure (Assfalg et al. 2008). We have addressed this problem by developing and validating standardized methods for both the management of participants and for urine sampling in food-based interventions in free-living individuals (Brownlee et al. 2010) and also for acute postprandial studies in a controlled environment (Fave et al. 2011; Lloyd et al. 2011b). Key features of these study protocols include behavioral restrictions, e.g. no alcohol and the consumption of a standardized meal in the evening before a clinic visit to provide an informative fasting urine sample immediately before the start of a dietary intervention. We hypothesized that a standardized evening meal would help provide a ‘normalized’ metabolic background against which differences in urine chemistry from either previous habitual dietary intake prior to clinic visit or in response to acute food intake during the test day could be detected (Fave et al. 2011; Lloyd et al. 2011b; Scalbert et al. 2009; Walsh et al. 2006). We tested that hypothesis in the current study in which participants followed a 3-day meal plan twice; on one occasion a standardized evening meal was consumed on the Pre-day whilst on the other occasion a low polyphenol meal of the participant’s choice was consumed. We observed that the majority of markers appeared discriminatory after both the standardized ‘normalizing’ meal and the low polyphenol meal of the participant’s choice, suggesting that the use of a strictly controlled standard evening meal immediately before a food intervention trial is not essential for biomarker discovery. However, we do not know whether removing all restrictions on the choice of evening meal on the Pre-day would provide satisfactory fasting/FMV samples for subsequent biomarker discovery. The standard evening meal was designed to be low in polyphenols: it is possible that this had a similar effect to that of the self-chosen low polyphenol meal against which it was tested. This remains to be investigated.

### Biomarker generalizability assessment using different food formulations

During the intervention period, several foods were introduced multiple times using different preparation, processing and cooking methods and were provided in different portion sizes as appropriate (Food Standards Agency 1994). This provided an opportunity to determine whether previously identified biomarkers for specific foods were generalizable when each food was consumed after use of different processing or preparation methods. Here we consider four examples of food and biomarker combinations viz. tartaric acid for grape exposure (Garcia-Perez et al. 2016); rhamnitol for apple exposure (Posma et al. 2017); DHPPA glucuronide for wholegrain exposure (Bondia-Pons et al. 2013); and anserine for meat exposure (Dragsted 2010; Lloyd et al. 2011b). To illustrate the approach, Table 2 shows data for intake of grapes and grape products through the six experimental days and the utility of tartaric acid as a biomarker of grape exposure. Supplemental data 4 shows the complete data for all four exemplar foods and their corresponding biomarkers. Grapes were consumed at different meal times during the day as both white and red grape products, including: grape juice (pasteurized/heat-treated/concentrated), raisins (dried), wine (fermented), whole grapes, sparkling grape juice (carbonated drink). The composition of urine samples collected 2–3 h after each meal (post breakfast, post-lunch, and post-dinner) was compared with that of a fasting sample from earlier the same day and differences tested using Random Forest classification modelling (Table 2). In addition, we quantified the concentration of tartaric acid in FMV urine on the following day. Tartaric acid was discriminatory after the consumption of all grape product formulations (Table 2 and Supplemental data 4). After a large or medium portion exposure (i.e. grape juice/wine etc.) tartaric acid was discriminatory in the immediate and the next-but-one post-prandial urine sample (2–3 h after exposure), but when a smaller dose was consumed (e.g. a berry smoothie containing 10% grape juice) the biomarker did not appear discriminatory until the next-but-one urine collection (> 6 h post-prandial; Supplemental data 4). For the day when no grape products were consumed, the tartaric acid concentration in FMV urine on the following day was < 1 µg/mL compared with concentrations between 9.28 and 26.24 µg/mL after consumption of a range of types and portions sizes of grape-derived foods and beverages (Table 2).

**Table 2:** Provision of grapes and grape products in each meal/snack throughout 6 experimental days and the utility of tartaric acid as a biomarker of grape consumption in different food/beverage formulations

Experimental day	Meal	Food component	Grape consumption (g)	Portion size	AUC	Mean tartarate concentration in FMV µg/mL (± std error) of following day
1	Breakfast	No grape product	0	N/A	0.72	11.03 ± 1.79
1	Lunch	No grape product	0	N/A	0.56	
1	Dinner	Red wine	200	Large	**0.93**	
2	Breakfast	Red berry smoothie (10% grape)	31	Small	0.79	26.24 ± 4.38
2	Lunch	No grape product	0	N/A	**0.94**	
2	Dinner	Raisins	43	Medium	*0.87*	
3	Breakfast	No grape product	0	N/A	*0.87*	15.8 ± 2.24
3	Lunch	No grape product	0	N/A	**0.95**	
3	Dinner	White wine	201	Large	*0.88*	
4	Breakfast	No grape product	0	N/A	0.67	0.70 ± 0.14
4	Lunch	No grape product	0	N/A	0.62	
4	Dinner	No grape product	0	N/A	0.74	
5	Breakfast	Red grape juice	204	Medium	**0.91**	10.44 ± 1.31
5	Lunch	Red grapes	125	Medium	**0.98**	
5	Afternoon snack & dinner	Red grapes & Red grape juice	208	Large	1	
6	Breakfast	White grape juice	204	Medium	*0.82*	9.28 ± 1.11
6	Lunch	White grapes	125	Medium	**0.94**	
6	Afternoon snack & dinner	White grapes & white grape juice	228	Large	**0.93**	

Menu plans illustrate the grape content of successive meals on each of 6 experimental days. For a signal/biomarker to appear discriminatory, the following thresholds were implemented: Random Forest (RF) Importance scores > 0.002, P-values < 0.05, area under the receiver operator characteristic (ROC) curve (AUC) 0.9 (highlighted in bold). Lower thresholds indicated putative discriminatory biomarkers (RF Importance scores > 0.001 < 0.002, AUC > 0.8 < 0.9) and deemed worthy of investigating further (highlighted in italics)

Tartaric acid occurs in grapes at relatively high concentrations (Ribéreau-Gayon et al. 2006; Velioglu 2009) and has been identified as a biomarker of wine or grape/grape juice consumption (Garcia-Perez et al. 2016; Regueiro et al. 2014). Our data suggest that it may be a generic biomarker of intake of all grape-derived foods and beverages. Urinary excretion of tartaric acid peaks between 4 and 8 h post consumption, the majority of the excretion occurs in the first 12 h and urinary concentrations decline close to baseline after 24 h (Garcia-Perez et al. 2016). This continued excretion of tartaric acid for 12 + h after grape product consumption explains why tartaric acid concentration remained relatively high (10.44 µg/mL) in the FMV following multiple intakes of grape juice on experimental day 5 and this marker was less discriminatory in the post-breakfast urine on the last study day. Additionally the formulation of grape product may also have influenced the concentration of tartaric acid measured in the FMV after the menu day. It has previously been shown that raisins have a higher concentration of tartaric acid than wine and grapes (Spiller and Spiller 2001) and grape juice has a lower concentration than wine and grapes due to the detartration process (Kodama et al. 2001; Soyer et al. 2003), which is reflected in our results (Table 2).

In this study, apple was consumed on two experimental days as a whole fruit, as a component of a mixed berry smoothie, juice (from concentrate), and within a cooked, sweetened pie. Rhamnitol appeared discriminatory after exposure to a medium portion size of all apple-containing food formulations and at a high concentration in the FMV the following day and was absent from urine collected after the consumption of meals not containing apple (Supplemental data 4) in agreement with previously published literature (Posma et al. 2017). However, the marker did not appear discriminatory after consuming a single smaller portion of apple in a pie (27 g apple) which suggests that this putative biomarker may not be sufficiently sensitive for detecting consumption of low intakes of this fruit.

Participants consumed wholegrain as rye bread (with and without a sourdough starter, toasted and untoasted), wholegrain bread (with and without kibbled malted wheat, toasted and untoasted), porridge oats (microwaved with milk) and wholegrain pasta (extruded). DHPPA glucuronide was a discriminatory biomarker for wholegrain foods, in general, but had stronger discriminatory power for toasted wholegrain bread and wholegrain pasta (Supplemental data 4) and was less explanatory for porridge oats consumption. Based on RF feature selection, several previously reported urine metabolites were highly ranked as putative biomarker candidates for wholegrain consumption, but many also correlated with recent exposure to other non-wholegrain foods (data not shown), and were deemed not to be wholegrain-specific. In agreement with published literature (Bondia-Pons et al. 2013), DHPPA glucuronide emerged as a discriminatory biomarker specifically for wholegrain exposure in this study. However, DHPPA glucuronide appeared to be a stronger discriminatory marker for exposure for some heat/thermally treated processed wholegrain foods such as wholegrain pasta, a thermally extruded wholegrain product and toasted bread when compared with untoasted bread, bread rolls and porridge oats.

Glucuronidated DHPPA was a poor marker on experimental day 6 after a lead-in day (experimental day 5) where there had been no consumption of wholegrain products and two medium portions of wholegrain on experimental day 6. It would have been expected that glucuronidated DHPPA concentration in the FMV of day 6 to be the lowest for the whole experimental period (instead it was higher than for experimental day 3-supplemental data 4). On that basis it should have provided a better test of the utility of the putative biomarker after consumption of the wholemeal rolls on experimental day 6. DHPPA is a metabolite of alkylresorcinols originating from the wholegrain bran fraction (Landberg et al. 2008). A potential limitation of our analysis is that for all of the wholegrain foods used in this intervention, information on the wholemeal wheat/rye flour content and ultimately the bran content was obtained from the food packaging, so there could potentially be an error associated with this.

Anserine proved to be a marker of consumption of salmon, cod and chicken (Supplemental data 4), but not of canned tuna, shellfish (prawns) or of red meat (beef, pork). There was a dose-dependent relationship (R^2^ = 0.62, Supplemental data 5) between consumption of poultry and fish (not including shellfish) and anserine concentration in FMV urine on the day following consumption of the test food. Anserine is found at relatively high concentrations in the muscle of many oily fish species (Abe 1983; Abe et al. 1993) and we have reported greatly elevated concentrations of anserine in urine after consumption of smoked salmon (Lloyd et al. 2011b). However, anserine was not discriminatory after consumption of canned tuna and prawns, despite much research suggesting that high levels can be found in these meats (Jones et al. 2011). The fact that anserine did not appear discriminatory after red meat (beef, pork) consumption was expected because these foods contain relatively low concentrations of anserine (Aristoy and Toldrá 2004; Dragsted 2010).

Overall, despite only investigating a limited number of different formulations of foods it was clear that some biomarkers can be markers of food consumption only when prepared in a certain way, while others can be food-specific biomarkers, independent of the processing/cooking method. Multiple markers or a panel of markers for food groups/unique foods would need to be developed to overcome these issues, while continuing to discover and develop unique food group biomarkers.

### Relationships between quantities of foods/beverages consumed and urinary concentrations of putative biomarkers

**Fig. 1:**
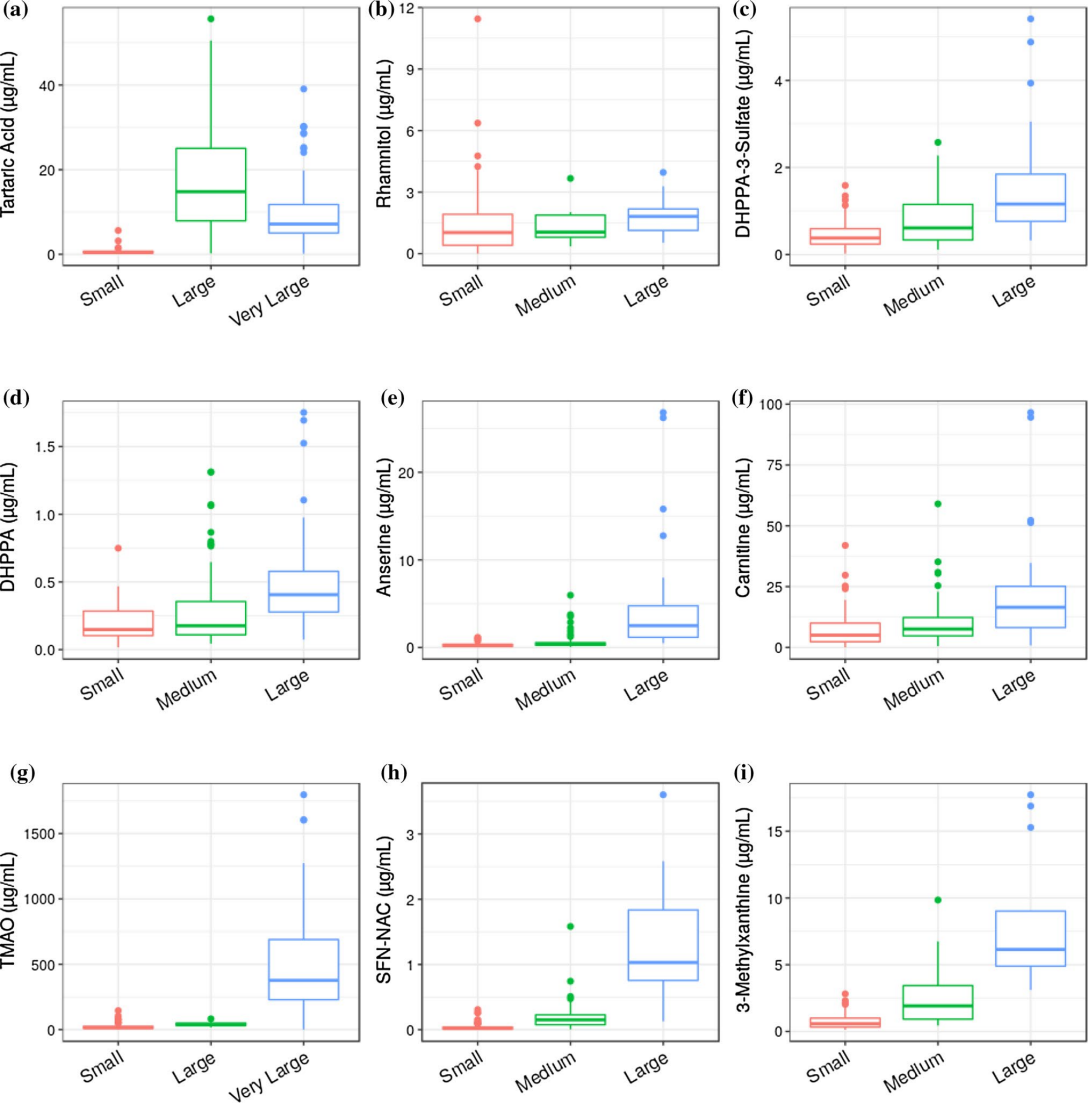
Boxplots of absolute concentrations of selected biomarkers in refractive index adjusted FMV urine the day after consumption of the test foods against portion size of foods/beverages consumed. **a** Tartaric acid for grape; **b** Rhamnitol for apple; **c** DHPPA-3-Sulfate for wholegrain; **d** DHPPA for wholegrain; **e** Anserine for poultry/fish; **f** Carnitine for red meat; **g** TMAO for fish **h** SFN-NAC: d,l-Sulforaphane-*N*-acetyl-l-cysteine for brassica; **i** 3-methylxanthine for cocoa. Significance values (*t* test) and Spearmans co-efficients are shown in supplemental data 5a

In the MAIN Study participants consumed different portion sizes of several foods^(18,19)^ that were categorized as small, medium, large and very large, based on UK FSA portion sizes (Food Standards Agency 1994). Although the kinetics of individual biomarker excretion differ and may be influenced by the food from which each metabolite is derived (Dragsted et al. 2018), for this comparison we opted to measure concentrations in FMV urine samples only since this spot urine type may be particularly useful for simultaneous assay of a comprehensive range of food intake biomarkers. In general, urinary concentrations of putative biomarkers increased with greater portion sizes of the test food/beverage (Fig. 1). As grape-product portion sizes increased from ‘small’ the absolute concentration of tartaric acid increased significantly too (Fig. 1a and Supplemental data 4 and 5a). Tartaric acid can undergo bacterial digestion, and it has been proposed previously that there are inter-individual differences in tartaric acid excretion due to differences in gut microbiota between individuals (Garcia-Perez et al. 2016). Apple products were consumed on only two experimental days and on one of these days a different formulation was consumed at each main meal to achieve a ‘large’ level of exposure; rhamnitol levels in the next day FMV were recorded as 1.85 µg/mL (Supplemental data 4). A FMV sample following a day when no apple products were consumed provided a ‘small’ exposure level (i.e. < 1 portion per day) and rhamnitol levels were found to be 0.52 µg/mL. Rhamnitol levels also exceeded 1.00 µg/mL in FMV samples following days with no expected exposure to apple products, however a more detailed investigation of processed foods consumed on these days often indicated apple products as an ingredient (data not shown). These factors probably explain why only a weak trend in the increase in concentration of rhamnitol was observed as the portion sizes increased, resulting in an insignificant *t* test statistic and a Spearmans co-efficient of only 0.18 (Fig. 1b and Supplemental data 5a).

The aglycone of DHPPA and the biotransformation product, DHPPA sulfate, were both available commercially as chemical standards and used to examine wholegrain exposure. Both of these markers demonstrated a good correlation (Supplemental data 5a; 0.49 and 0.59 respectively) with wholegrain exposure (Fig. 1c, b). The absolute concentrations of anserine in FMV urines correlated well (Supplemental data 5a; 0.62) against the portion size of poultry and oily fish consumed the day before (Fig. 1e). Carnitine is present at high concentrations in red meat (Dragsted 2010) and its concentration in FMV urine showed a good correlation with red-meat portion size consumed the previous day (Fig. 1f). TMAO (Trimethylamine-N-oxide) is a published fish marker (Dragsted 2010; Lloyd et al. 2011b) and showed a significant increase in concentration as the portion size increased (Fig. 1g and Supplemental data 5a). It has been shown recently that chronic red meat exposure can cause a small increase in urinary TMAO due to gut microbial production from carnitine (Wang et al. 2018) however sufficient detail was not provided to determine if fish products were included in the menu design; urinary TMAO levels were much lower than those reported after fish exposure in the present study.

To broaden the range of investigated food groups we quantified other published markers representing brassica (SFN-NAC: D,L-Sulforaphane-N-acetyl-l-cysteine (Andersen et al. 2013)) and cocoa (3-methylxanthine (Llorach et al. 2013)). Both markers also showed an increase in absolute concentration in urine samples as the portion size increased (Fig. 1h–i). To take into account relative differences in fluid intake between participants all FMV urine samples were normalized by refractive index prior to analysis to ensure all MS measurements were made within a similar dynamic range. These data however do not take into account differences in the total volume of individual FMV urines which may account for some of the variability observed in Fig. 1. However after adjustment for FMV excreted volume (Supplemental data 5b), the same conclusions are made with regards to the Spearman correlation and P-values for all biomarkers.

## Strengths and limitations of study

The data presented focus on determining the utility of the food intervention study design and the urine sampling strategy for confirming or refuting the usefulness of putative biomarkers for a range of related foods and beverages. A major strength of the study design was the desire to expose participants simultaneously (and for some foods multiple times) to a comprehensive range of foods representing the main components of their habitual diet, rather than providing foods as isolated single interventions. This approach offered opportunity to confirm the ability of putative biomarkers to report exposure to specific foods/food groups in the same study population against a variable background of exposure to other foods. Likewise, many target foods were presented in multiple formulations, including the use of different processing methods. Combined with the fact that participants prepared and consumed all meals and collected samples in their own home, these food intervention design attributes allowed us to examine biomarker performance under conditions of much more realistic simulated habitual eating behavior. From a practical perspective it is worth noting that in some cases the best candidate markers are likely to be sulfate or glucuronides but surrogate biomarkers may need to be selected for future quantification and use in monitoring nutrition due to lack of commercial sources or cost considerations.

## Concluding remarks

We designed and tested a dietary intake biomarker discovery and validation strategy based on a comprehensive food intervention study involving free-living individuals who prepared and consumed the test meals and collect urine samples at home. The demonstration of the utility of spot urine samples, in combination with robust dietary biomarkers to report multiple diet components (despite major sources of variance) will allow the future validation of dietary biomarker technology in epidemiological studies. We feel that multiple, well-spaced spot samples collected over several weeks would be able to capture habitual exposure to a wide range of food groups. The ultimate aim of our studies is to deploy a comprehensive biomarker panel able to aid in monitoring habitual dietary exposure in clinical trials or population surveys at a range of scales.

## Electronic supplementary material

Below is the link to the electronic supplementary material.

Supplementary material 1 (XLSX 38 kb)

## Data Availability

Metabolomics data have been deposited to the EMBL-EBI MetaboLights database with the identifiers MTBLS928 (quantification data) and MTBLS929 (FIE-HRMS data). The complete datasets can be accessed here: https://www.ebi.ac.uk/metabolights/MTBLS928 and https://www.ebi.ac.uk/metabolights/MTBLS929 respectively.
